# Initial Clinical Evaluation of the Modular Prosthetic Limb

**DOI:** 10.3389/fneur.2018.00153

**Published:** 2018-03-19

**Authors:** Briana N. Perry, Courtney W. Moran, Robert S. Armiger, Paul F. Pasquina, Jamie W. Vandersea, Jack W. Tsao

**Affiliations:** ^1^Walter Reed National Military Medical Center, Bethesda, MD, United States; ^2^Johns Hopkins University’s Applied Physics Laboratory, Laurel, MD, United States; ^3^Uniformed Services University of the Health Sciences, Bethesda, MD, United States; ^4^Advanced Arm Dynamics, Redondo Beach, CA, United States; ^5^University of Tennessee Health Science Center, Memphis, TN, United States

**Keywords:** upper limb amputation, upper extremity prosthesis, Modular Prosthetic Limb, surface electromyography, pattern recognition control, virtual integration environment, traumatic amputation, neurorehabilitation

## Abstract

The Modular Prosthetic Limb (MPL) was examined for its feasibility and usability as an advanced, dexterous upper extremity prosthesis with surface electromyography (sEMG) control in with two individuals with below-elbow amputations. Compared to currently marketed prostheses, the MPL has a greater number of sequential and simultaneous degrees of motion, as well as wrist modularity, haptic feedback, and individual digit control. The MPL was successfully fit to a 33-year-old with a trans-radial amputation (TR01) and a 30-year-old with a wrist disarticulation amputation (TR02). To preserve anatomical limb length, we adjusted the powered degrees of freedom of wrist motion between users. Motor training began with practicing sEMG and pattern recognition control within the virtual integration environment (VIE). Prosthetic training sessions then allowed participants to complete a variety of activities of daily living with the MPL. Training and Motion Control Accuracy scores quantified their ability to consistently train and execute unique muscle-to-motion contraction patterns. Each user also completed one prosthetic functional metric—the Southampton Hand Assessment Procedure (SHAP) for TR01 and the Jebsen-Taylor Hand Function Test (JHFT) for TR02. Haptic feedback capabilities were integrated for TR01. TR01 achieved 95% accuracy at 84% of his VIE sessions. He demonstrated improved scores over a year of prosthetic training sessions, ultimately achieving simultaneous control of 13 of the 17 (76%) attempted motions. His performance on the SHAP improved from baseline to final assessment with an increase in number of tasks achieved. TR01 also used vibrotactile sensors to successfully discriminate between hard and soft objects being grasped by the MPL hand. TR02 demonstrated 95% accuracy at 79% of his VIE sessions. He demonstrated improved scores over months of prosthetic training sessions, however there was a significant drop in scores initially following a mid-study pause in testing. He ultimately achieved simultaneous control of all 13 attempted powered motions, and both attempted passive motions. He completed 5 of the 7 (71%) JHFT tasks within the testing time limit. These case studies confirm that it is possible to use non-invasive motor control to increase functional outcomes with individuals with below-elbow amputation and will help to guide future myoelectric prosthetic studies.

## Introduction

By the year 2050, an estimated 3.6 million persons will be living with amputations within the United States (1). Military operations in Iraq and Afghanistan have led to 1716 United States Military Service members sustaining major limb loss as of September 2017, with 297 (17.3%) losing an upper limb (J. C. Shero, personal communication, 10/03/2017). Despite advances in upper limb prostheses, there continues to be a high rate of user abandonment (2). Currently, the most sophisticated myoelectric prostheses are controlled by up to six surface electromyography (sEMG) electrodes offering the user a maximum of 3° of sequential movement.

**Figure 1:**
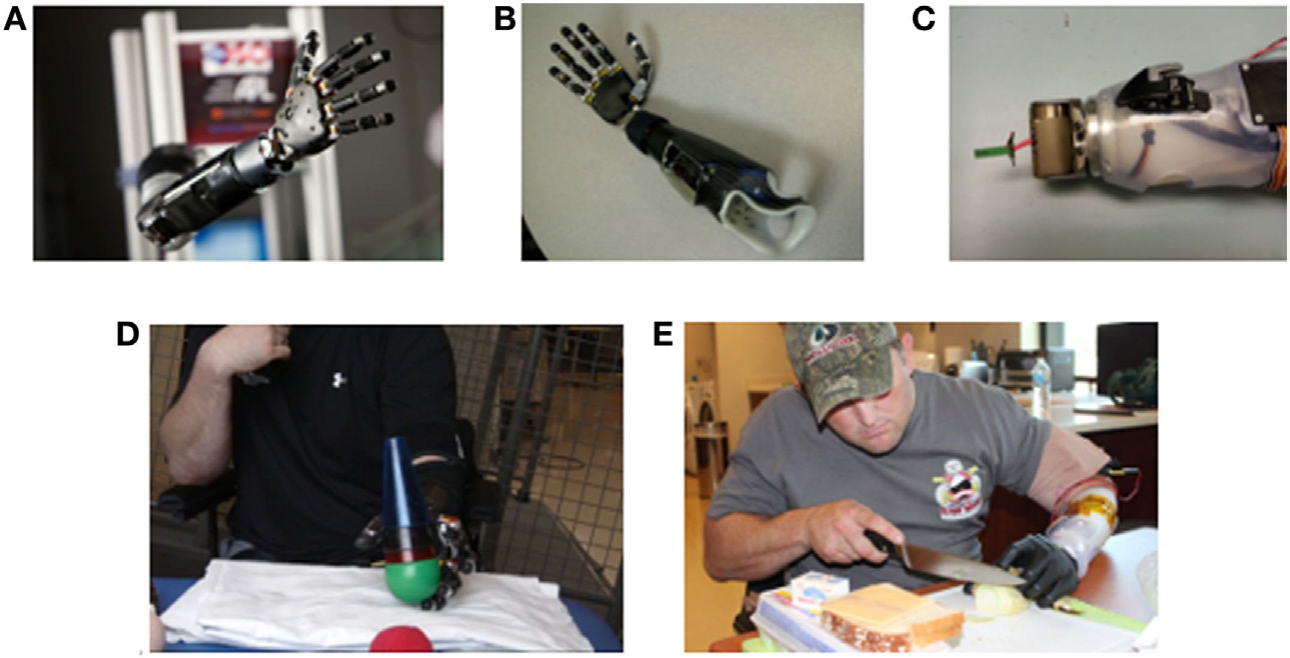
Images of Modular Prosthetic Limb (MPL) fitting and training by users with upper limb amputation. **(A)** The MPL configured at shoulder level with integration of all sensory, motor, and control capabilities. **(B)** The trans-radial MPL configuration for TR01. **(C)** The modulation of the MPL wrist to one degree of freedom for TR02 to support proper anatomical arm length and facilitate completion of activities of daily living. **(D)** TR01 performing reach, grasp, and manipulation tasks during a clinical use session. **(E)** TR02 performing a cooking task at Walter Reed National Military Medical Center in Bethesda, MD, USA.

The Modular Prosthetic Limb (MPL) was developed through the DARPA Revolutionizing Prosthetics Program to provide up to 26 articulating degrees of freedom (DOF) *via* 17 actuators from shoulder to hand and sensory feedback *via* vibrotactile sensors (Figure 1A) (3). When configured at the below-elbow level, the MPL has 10 actuators of hand motion and up to three DOF of powered wrist motion. The MPL offers many improvements over existing prosthetic systems, such as increased speed, increased motions, wrist modularity, haptic feedback, and individual digit control (4). A traditional two-site, myoelectric prosthesis offers the user only two distinct wrist motions (one wrist DOF) and hand open/close, while the MPL offers up to six distinct wrist motions (three wrist DOF), hand open, six unique hand grasps, and digit control.

Herein, we describe two case studies with the MPL. A 33-year-old with a left trans-radial amputation (TR01) and a 30-year-old with a left wrist disarticulation amputation (TR02) underwent MPL fittings and socket fabrication after demonstrating control within the virtual integration environment (VIE) (5–7). Participants completed a variety of clinical sessions and functional metrics with the MPL. Due to the restricted availability of TR02, the case protocols differ. These cases are the first to demonstrate the feasibility of using non-invasive means to provide advanced myoelectric prosthetic control to individuals with below-elbow amputations.

## Methods

### Participants

TR01 sustained a left trans-radial amputation 7 months prior to study participation. TR02 sustained a left wrist disarticulation amputation 10 months prior to study participation. Both individuals are active duty males who sustained their injuries in the line of duty from improvised explosive devices. Written informed consent was obtained from the participants for the publication of this case report. Both participants denied phantom limb and residual limb pain.

### Prosthetic Fitting

We utilized a standard TRAC self-suspending socket design for socket casting (8). Eight non-invasive LTI dome electrode pairs (Liberating Technology, Inc. Holliston, MA, USA) and one ground electrode transduced sEMG signals. In conventional direct control myoelectric prostheses, each pair of electrodes maps to a single input channel; however, we created a wired array of input channels to enable eight-channel pattern recognition control. Electrodes were placed in a flexible, Proflex with Silicone socket (Cascade Orthopedic Supply, Inc. Chico, CA, USA) (9). EMG signals were sampled at 1 kHz, filtered at 15 Hz with a third-order Butterworth high-pass filter, and processed at 50 Hz allowing for a new motion to be generated every 20 ms.

For TR01, a self-suspending laminated endoskeletal double wall socket with flexible inner liner was fabricated. A custom-made piece housed the processing boards and facilitated prosthetic attachment (Figure 1B). For sensory feedback, two additional LTI dome electrodes backed with coin style vibromotors [Precision Microdrive C08-001 (London, UK)] were embedded within the socket and used as closed loop sensory feedback actuators. When the MPL hand grasped an object, it triggered joint torque sensors in the prosthetic fingers to transmit a vibratory signal to the residual limb.

For TR02, a double wall thermoplastic socket was fabricated. To accommodate the longer residual limb (28.5 cm), the boards were housed along the wall of the socket rather than at the wrist-end. A temporary, rigid thermoplastic frame housed the electronics (Figure 1C). An Upper-Ex locking liner (Ossur, Reykjavik, Iceland) and ratchet lanyard suspension system (10) adhered to the middle of the limb, and an adjustable ratchet strap exited the socket distally. TR02 opted out of sensory feedback integration due to his desire to first master motor control.

### Wrist Modularity

The modularity of the MPL wrist allows for the accommodation of limb length. With a shorter residual limb, TR01 could wear a wrist with three-powered DOF (flexion/extension, supination/protonation, radial/ulnar deviation) without deviating from his anatomical limb length. With a longer residual limb, TR02 was provided with a wrist with one-powered (flexion/extension) and one-passive (supination/protonation) DOF (Figure 1C). MPL wrist lengths for TR01 and TR02 measured 28 and 19 cm, respectively.

### Virtual Training

Both participants began training with pattern recognition control within the VIE—a software system for learning and evaluating prosthetic use created by the Johns Hopkins University’s Applied Physics Laboratory (5–7). Using eight sEMG electrode pairs, participants trained the computer to recognize their unique muscle-to-motion patterns and practiced controlling the upper limb of a virtual avatar. VIE sessions were assessed using the Motion Control test, which challenges the user to recreate their trained muscle patterns in response to prompted motions.

### Clinical Training

TR01 completed 16 clinical training sessions (each 60–90 min) providing for a total of 20 training hours over 12 months. Each session began with a basic set (hand open, spherical grasp, wrist flexion/extension, wrist pronation/supination). Additional motions were added based on user feedback and demonstrated motor control. He practiced using the MPL to complete activities such as cone stacking and ball lifting (Figure 1D). Each session ended with a Motion Control test (11, 12).

TR02 completed nine clinical training sessions (each 60 min) providing for a total of nine training hours over 6 months. These sessions followed a similar pattern to those of TR01. The difference in training time between participants was due to TR02’s departure from WRNMMC.

### Training Interface

Typical systems for prosthetic control rely on supervised machine learning where the user is presented with a pre-programmed set of visual prompts. TR01’s clinic sessions began with such a system, but feedback early on led us to conceptualize a novel training interface where he could drive the data collection process. Using a standard gaming controller, he selected which motions were trained and for how long data was collected. The training algorithm was re-computed every 10 muscle-to-motion pattern recordings. This system was implemented on TR01’s sixth training session and used throughout all sessions with TR02.

### Assessments

The Motion Control test—an early version of the one DOF Target Achievement Control metric—was used to assess pattern recognition control (11, 13). The test generates a Training Accuracy score by recording a user’s unique muscle-to-motion contraction patterns and a Motion Classification Accuracy Score by assessing his ability to recreate these patterns. The test occurs within the VIE interface with the participant wearing the MPL. Scores represent the number of motions achieved divided by the number of motions attempted. For a motion to be achieved, 10 correct and consecutive motion classifications are required within a 5-s window. Motion sets were defined as “basic” (4–5 motions), “intermediate” (6–7 motions), and “advanced” (10–12 motions). Response times represent the average time passed from selection of the motion to completion of 10 consecutive classifications.

Currently, there is no gold standard for the evaluation of myoelectric prosthetic use. We based metric selection upon the recommendations of the upper limb prosthetic outcome measures (UPLOM) and similar studies of dexterous prosthetic arms (14–16). To assess TR01’s function with the MPL, he completed the abstract light object portion of the Southampton Hand Assessment Procedure (*SHAP*) at his first and final sessions (17, 18). The SHAP involves transfer of a single object using various grasps. We chose this assessment because it utilized multiple grasp patterns and the MPL configuration for TR01 utilized many DOF of wrist motion. For TR02, we used the Jebsen-Taylor Hand Function Test (*JHFT*), which he completed with the MPL, his conventional myoelectric prosthesis, and his intact limb at his final session (Figure 1E) (19). We chose the JHFT for TR02 because it focuses on simulating ADLs.

Both participants contributed subjective feedback on an ongoing basis. TR02 additionally completed the Trinity Amputation and Prosthesis Experience Scales-Revised (*TAPES-R*) (20).

## Results

### Case 1: TR01

#### VIE Training

TR01 completed 20 VIE sessions (each 30 min) between June and September 2012. For a basic motion set, he achieved greater than 95% accuracy at 16 of 19 assessments (84%) with a mean accuracy score of 97.6%. The threshold for prosthetic efficiency was defined as 95% accuracy based on findings from internal pilot studies with the MPL. TR01 achieved 100% accuracy with the basic motion set when using his intact (i.e., control) limb at four assessments.

#### Motions Achieved

TR01 achieved performance of 13 independent motions: hand open, wrist flexion/extension, wrist pronation/supination, wrist radial/ulnar deviation, spherical/fine pinch grasps, and articulation of four digits. For comparison, only four discrete motions can be achieved with a conventional prosthesis (hand open/close, wrist pronation/supination). He attempted but was unable to perform four motions: cylindrical/pointer/lateral grasps and ring finger articulation. Of note, TR01 reported that his phantom ring finger was “frozen” both before and throughout the study. To facilitate completion of ADLs, clinical use sessions focused on the following motions: hand open, spherical/fine pinch grasps, wrist flexion/extension, and wrist radial/ulnar deviation.

#### Accuracy Scores

Clinical use of the MPL by TR01 fell into three time intervals occurring at 1 month (5 sessions), 6 months (10 sessions), and 12 months (5 sessions). During the 60-to-90-min sessions, TR01 trained motions based on daily task selection. Training Accuracy scores improved across sessions with means of 84.5, 89.8, and 91.0% at months 1, 6, and 12, respectively. Motion Control Accuracy scores also increased over time ranging from 31 to 83% for the basic set and 20–52% for the intermediate set. Motion Control Accuracy scores increased within each session grouping and across the study, but there was an initial decrease in scores at the start of each new session grouping. At the 12-month grouping, Motion Control Accuracy scores for an advanced set ranged from 41.2 to 65%. Average motion completion time was 1.39 ± 0.45 s.

#### Functional Assessment

**Figure 2:**
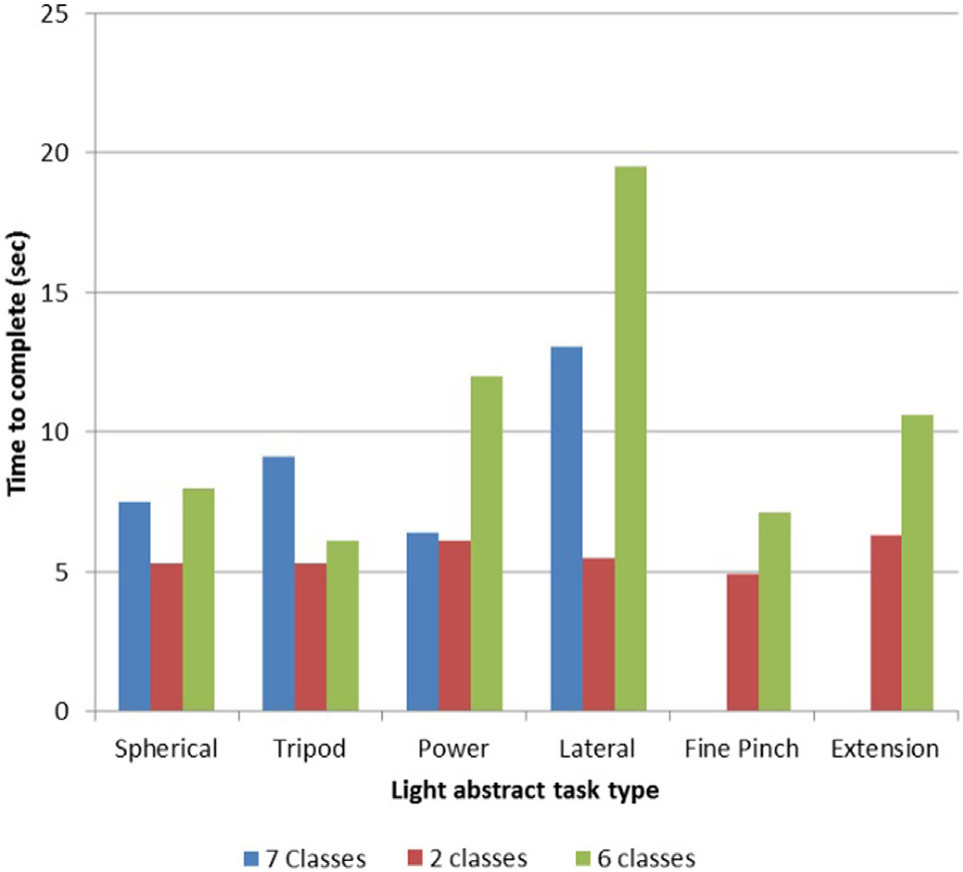
Light object Southampton Hand Assessment Procedure (SHAP) results for TR01. TR01 demonstrated scalable control of the Modular Prosthetic Limb while completing the light object *SHAP* (13, 20) using sets of two, six, and seven simultaneously controllable motion classes. Completion times were lowest with the two-motion set, which included the motions of hand open and spherical grasp. The six-motion set added wrist flexion/extension and wrist protonation/supination, while the seven-motion set included fine pinch grasp.

TR01’s performance of the light abstract object portion of the *SHAP* revealed a training time effect, as with more experience he achieved more tasks and completed tasks quicker. With a two-motion set (hand open, one grasp), he completed all six tasks with a mean time per task of 5.50 s (Figure 2). This result is what is expected when using a myoelectric prosthesis with a passive wrist and open-and-close hand (14). With a seven-motion set, he initially completed four of six tasks with a time of 9.02 s, but later completed all six tasks with a time of 10.50 s (Figure 2).

#### Tactile Feedback

TR01 utilized tactile feedback in the form of pressure discrimination during one clinical session. When grasping an object with the prosthetic hand, he felt a proportional vibration on his residual limb that allowed him to successfully differentiate between hard and soft objects.

#### User Feedback

TR01 felt confident that more practice with the MPL would lead to improved control. He thought the MPL was “more natural” to use than his conventional prosthesis (Appendix contains conventional prosthetic information). He did not feel that the MPL plus battery weight (1.71 kg/3.78 lb + 0.38 kg/0.84 lb) was problematic compared to his conventional prosthetic weight (0.98 kg/2.15 lb).

### Case 2: TR02

#### VIE Training

TR02 completed 20 VIE sessions (each 30 min) from September to October 2012. He achieved greater than 95% accuracy with the basic set at 23 of 29 assessments (79%) with a mean score of 97.4%. He achieved greater than 95% accuracy with the intermediate set at 7 of 14 assessments (50%), but with an accuracy score less than the target 95% (92%). He achieved 100% accuracy with the basic set when using his intact (i.e., control) limb at eight assessments.

#### Motions Achieved

TR02 achieved performance of all 13 attempted powered motions: hand open, wrist flexion/extension, cylindrical/spherical/fine pinch/pointer/lateral grasps, and articulation of five digits. He achieved control of both available passive motions: wrist pronation/supination. In comparison, a conventional prosthesis has only four discrete motions (hand open/close, wrist pronation/supination). At clinical sessions, he preferred to practice with hand open, wrist flexion/extension, and spherical/cylindrical grasps.

#### Accuracy Scores

Clinical use of the MPL by TR02 fell into two time intervals consisting of six and three sessions and divided by a 2-month gap due to user availability. Training Accuracy scores averaged 93.3% across sessions. Motion Control Accuracy scores for a basic set increased from 68 to 90% across the first six sessions and from 30 to 73% across the final three sessions. The 2-month clinical pause between the two session groupings corresponded to a decline in scores from 90 to 30%. Motion Control Accuracy scores for an intermediate set varied from 37 to 52%. With the advanced set, the maximum accuracy score achieved was 61%. Average motion completion time was 1.40 ± 0.24 s.

#### Functional Assessment

TR02 successfully completed five of the seven (71%) *JHFT* tasks within the 2-min test limit (Table 1). Times with the MPL were slower than with his conventional myoelectric prosthesis and times with both prostheses were slower than with his intact, dominant limb. With his conventional prosthesis and his intact limb, he completed all tasks within the time limit. With the added dexterity of the MPL, consistently and precisely bringing the fingers together for small object manipulation proved challenging (Table 1).

**Table 1:** Jebsen-Taylor Hand Function Test (*JHFT*) results for TR02.

Task	MPL		Conventional myoelectric		Normative data		Comparison data	
			
	Non-dominant	Dominant	Non-dominant	Dominant	Non-dominant	Dominant	Multifunctional myoelectric	Conventional myoelectric
Writing	46.18	14.97	30.71	15.71	32.3	12.2		
Simulated page turning	100.15	4.88	14.11	5.12	4.5	4		
Lifting small common objects	*120*	7.07	31.53	6.76	6.2	5.9		
Simulated feeding	23.53	8.51	13.51	9.52	7.9	6.4		
Stacking checkers	*120*	4.37	25.6	3.65	3.8	3.3		
Lifting large light objects	48.5	3.25	8.36	3.21	3.2	3		
Lifting large heavy objects	52.91	3.19	6.65	3.25	3.1	3		
Total times	511.27	46.24	130.47	47.22	61	37.8	325	224

*TR02 completed the *JHFT* with his non-dominant, left, amputated limb using both the Modular Prosthetic Limb (MPL) and his conventional myoelectric prosthesis. The MPL wrist was configured to have four powered (hand open, spherical grasp, wrist flexion/extension) and two passive (wrist pronation/supination) degrees of freedom (DOF). For a control, he completed the tasks with his right, intact, and dominant limb. Completion times are in seconds. Overall, TR02 successfully completed five of the seven (71%) tasks within the 2-min time limit using the MPL. The tasks that he did not complete within the time limit are italicized. It took him the longest to complete fine motor tasks that required the prosthetic fingertips to touch. With his conventional myoelectric prosthesis he was able to complete all seven tasks within the testing time limit and with shorter times than it took to complete the tasks with the MPL. He completed all tasks more quickly with his right, intact, and dominant limb than he did with either the MPL or his conventional prosthesis. Normative data for task completion times with able-bodied males from 20 to 59 years old are provided (16). Comparison data are also given for persons with limb amputation completing the task with either a multifunctional prosthesis (i.e., 4 DOF) or a conventional myoelectric prosthesis (i.e., 2 DOF) (14)*.

#### User Feedback

In the *TAPES-R* survey, TR02 reported that his activities were less restricted by the MPL than by his conventional myoelectric prosthesis, but that he was better adjusted to and more satisfied with his conventional prosthesis. He was most satisfied by the comfort of the MPL and least satisfied by its weight (1.62 kg/3.58 lbs plus battery weight of 0.38 kg/0.84 lb compared to 0.95 kg/2.10 lbs for conventional prosthesis). His favorite MPL feature was the multi-finger usability. He wanted more practice with the MPL before using it to complete everyday tasks.

## Discussion

These case studies investigated whether the MPL could be utilized as a dexterous prothesis at the trans-radial and wrist disarticulation levels. For both cases, the MPL was operated by non-invasive, sEMG and pattern recognition control (3, 12, 21). The participants trained with the VIE before completing numerous clinical sessions and functional metrics with the MPL (5–7). Both cases provide valuable feedback on myoelectric prosthetic design and fitting and needed insight into advanced myoelectric prosthetic use by individuals with upper extremity amputation. The findings can be applied to future multi-participant, controlled prosthetic studies.

The first milestone was demonstrating the ability to integrate the highly dexterous capabilities of the MPL with current industry socket design. The successful fitting of the MPL to two individuals of differing arm length was completed while preserving individual limb length. Utilizing the wrist modularity feature of the MPL, we configured a three-powered DOF wrist for TR01 and a one-powered/one-passive DOF wrist for TR02. Wrist modularity is specific to the MPL.

The second milestone was demonstrating the ability to control the high number of simultaneous degrees of prosthetic motion. Both users successfully commanded up to 13 motions, representing a total of 17 motions. This is compared to current industry myoelectric prostheses which offer at most six motions. The feature of digit control is unique to the MPL.

Currently, there is no gold standard for the number of motions simultaneously commanded. Thus, we allowed users to select the motions they utilized at each session and for a given task. Both participants noted that access to a high number of motions improved their ability to complete ADLs. The results, however, suggest that control accuracy decreases as the number of available motions increases. This relationship was expected to some extent, as cognitive burden increases with more complex motion sets. Existing research suggests that increased training time would lead to improved control accuracy, as the reinforcement of muscle contraction patterns through consistent training paradigms correlates with improved performance of grasps (22). Future research is needed to elucidate how accuracy would improve with longer prosthetic training time, less interruptions between clinical use sessions, and at-home MPL use.

The third milestone was increasing the number and complexity of motions across sessions. The high Training Accuracy scores of both users represents effective training with pattern recognition control, while the increasing Motion Control Accuracy scores show an ability to retain and strengthen these skills over time. The functional application of these achieved motions was tested using a suite of prosthesis metrics adopted per UPLOM standards (14). Speed and functional output improved across months of clinical testing for both users. For TR02, JHFT results revealed that function with the MPL was inferior to function with his conventional myoelectric prosthesis. It is important to note that TR02 had significantly more experience with his conventional prosthesis (i.e., 1 year of daily use). Future studies would benefit from similar periods of prosthetic exposure to allow for better functional comparisons.

The fourth milestone achieved was the addition of haptic feedback to the MPL. TR01 experienced vibrotactile feedback against the surface of his residual forearm in response to grasping an object. The vibrotactile response increased relative to the force applied to the prosthetic fingertips allowing him to deduce the stiffness of objects being grasped (3). TR02 opted out of the use of haptic feedback, as he preferred to focus on training motor control.

There were occasional gaps between MPL testing sessions and, consequently, between exposures to pattern recognition control. During non-study days, both participants utilized passive prostheses and/or conventional myoelectric prostheses with two-site direct control. Research shows that consistent exposure to pattern recognition control results in the greatest improvements in motion selection accuracy, speed, and total number of motions controlled (21). Future studies should keep pattern recognition training consistent and limit the input of other control modalities.

Interestingly, both users indicated that changes in their phantom limb affected which motions they could intuitively achieve each day. For example, with an immobile phantom ring finger, TR01 could not develop a consistent signal for ring finger articulation. They expressed a strong desire to continue practicing with the MPL, which reflects a reduced risk of prosthetic abandonment (2, 23).

Together, these user experiences uniquely demonstrate early clinical operability with the MPL, which as the first non-invasively controlled advanced arm prosthesis holds the potential to dramatically advance clinical outcomes following upper limb loss.

## Ethics Statement

At the time of data collection, case studies were exempt from IRB processes at Walter Reed National Military Medical Center.

## Author Contributions

BP completed training with the VIE platform, assisted with the administration of clinical use sessions, and lead manuscript creation and submission. CM lead MPL training and clinical use sessions with TR02, managed MPL software, and contributed to manuscript creation, including figure generation; RA contributed to study design, lead MPL training and clinical use sessions with TR01, managed MPL and VIE software, and assisted with manuscript creation; PP organized study objectives, funding, and collaborations, and oversaw study execution; JV lead prosthetic socket creation and fittings and assisted with the manuscript writing; and JT oversaw study execution and lead manuscript editing.

## Conflict of Interest Statement

The opinions or assertions contained herein are the private views of the authors and are not to be construed as official or as reflecting the views of the Department of the Navy or the Department of Defense.

## Supplementary Material

TR01’s conventional prosthesis is a left trans-radial myoelectric prosthesis with a flexible Proflex inner socket and rigid laminated carbon fiber frame (Cascade Orthopedic Supply, Inc. Chico, CA, USA) and a Wrist Rotator, and a Vari-Plus hand with a PVC glove (Otto Bock, Duderstadt, Germany). It is controlled *via* 2 surface-mounted Otto Bock suction electrodes (13E202 = 60) placed on the forearm over either the wrist extensor or flexors. Suspension is achieved through a trans-radial supracondylar self-suspending TRAC socket with three-quarter modification.

TR02’s conventional prosthesis is a left wrist disarticulation myoelectric prosthesis with a flexible inner socket with a laminated frame, a Quick Disconnect wrist and Vari-Plus hand (Otto Bock, Duderstadt, Germany), an Upper-Ex silicone liner (Ossur, Reykjavik, Iceland), and an adjustable, Velcro lanyard suspension system. Control is achieved *via* 2 surface-mounted Otto Bock suction electrodes. The Velcro lanyard suspension system is affixed to the distal end of the Ossur Upper-Ex liner, exists the socket distally, and engages to a D-ring located on the medial aspect of the socket. The liner and suspension system are fit to a standard wrist disarticulation socket and preparatory Thermolyn (Otto Bock, Minneapolis, MN, USA) thermoplastic frame.

Movie S1 Executing spherical grasp to lift a ball.

Movie S2 Using fine pinch grasp to lift a block.

Movie S3 Demonstrating individual finger articulation.
